# Leveraging Interdisciplinary Teams to Develop and Implement Secure Websites for Behavioral Research: Applied Tutorial

**DOI:** 10.2196/19217

**Published:** 2020-09-23

**Authors:** Christie L Martin, Eydie N Kramer-Kostecka, Jennifer A Linde, Sarah Friend, Vanessa R Zuroski, Jayne A Fulkerson

**Affiliations:** 1 School of Nursing University of Minnesota Minneapolis, MN United States; 2 School of Kinesiology University of Minnesota Minneapolis, MN United States; 3 School of Public Health University of Minnesota Minneapolis, MN United States; 4 Office of Information Technology University of Minnesota Minneapolis, MN United States

**Keywords:** research ethics and compliance, website development, behavioral research, digital interventions, website authentication, website security

## Abstract

Behavioral researchers are increasingly using interactive digital platforms, either as standalone or supplementary intervention tools, to facilitate positive changes in research participants’ health habits. Research-oriented interactive websites optimally offer a variety of participatory mediums, such as blogs, user-driven content, or health activities. Owing to the multidirectional features of interactive websites, and a corresponding need to protect research participants’ identity and data, it is paramount that researchers design ethical platforms that ensure privacy and minimize loss of anonymity and confidentiality. Authentication (ie, digital verification of one’s identity) of interactive sites is one viable solution to these concerns. Although previous publications have addressed ethical requirements related to authenticated platforms, few applied guidelines in the literature facilitate adherence to ethical principles and legally compliant study protocols during all phases of research website creation (feasibility, design, implementation, and maintenance). Notably, to remain compliant with ethical standards and study protocols, behavioral researchers must collaborate with interdisciplinary teams to ensure that the authenticated site remains secure and usable in all stages of the project. In this tutorial, we present a case study conducted at a large research university. Through iterative and practical recommendations, we detail lessons learned from collaborations with the Institutional Review Board, legal experts, and information technology teams. Although the intricacies of our applied tutorial may require adaptations based on each institution’s technological capacity, we are confident that the core takeaways are universal and thus useful to behavioral researchers creating ethically responsible and compliant interactive websites.

## Background

In recent years, health behavior researchers have begun to use digital health promotion tools (eg, websites and mobile applications) as intervention platforms or supplemental tools due to the cost-effectiveness and availability of technology [1,2]. Many researchers are utilizing interactive web technologies with user-driven content and bidirectional participatory design features (eg, discussion boards, blogs, or gaming) to increase access to socially supportive digital systems that allow participants to network and engage with other participants online [3-7]. The interactive and collaborative design elements of these sites have the potential to enhance socially supportive environments aimed at facilitating healthy behavior change. However, the multidirectional features supported by interactive sites also introduce a level of inherent risk to the research participant: loss of anonymity and confidentiality [5-16].

In the United States (US), research institutions that receive government funding for human subjects research must abide by the tenets of the Common Rule, which are ethical standards upheld by Institutional Review Boards (IRBs) [17,18]. Behavioral researchers must submit protocols to be reviewed by the IRB to ensure that research projects are compliant with federal and institutional ethical rules and regulations. These rules and regulations include such things as informing participants of research risks and benefits, ensuring voluntary consent to participation, documenting all study protocols, and safeguarding participant data. Behavioral researchers who utilize electronic tools and mediums (eg, electronic health records) must also abide by Health Insurance Portability and Accountability Act (HIPAA) guidelines—information privacy standards set forth by the US Department of Health and Human Services—to protect identifiable information. Adhering to these standards helps to minimize the threat of disclosing protected health information (PHI; anything from social security numbers to medical record numbers) or personally identifiable information (PII; non-health specific personal identifiers, like phone numbers, license plate numbers, pictures, email addresses, etc) [10,12,19-21]. To conduct ethically responsible and legally compliant behavioral research aimed at protecting human subjects, investigators often interface with ethics (ie, IRB) and compliance (ie, legal and compliance officers) support teams. Adhering to ethics and compliance standards are particularly important for behavioral researchers utilizing interactive websites as health promotion tools, as these researchers must carefully consider how to simultaneously limit open access to website materials and protect research participants’ PHI/PII.

A viable solution to protecting participants’ PHI/PII before they engage with research websites is to create authenticated logins. To facilitate this procedure, behavioral researchers can collaborate with information technology (IT) support teams. In human subjects research, authentication—digital verification of one’s identity—involves the use of a de-identified username and password. Unfortunately, however, best practice ethics and compliance guidelines that clearly outline practical recommendations for when or how to implement digital authentication processes are currently lacking in the literature. In 2019, the Association of Internet Researchers’ ethics committee released the *Internet Research: Ethical Guidelines 3.0*. These guidelines highlight the importance of utilizing closed-access digital platforms to enhance security, yet their overarching considerations for protecting research participants’ anonymity and privacy are quite broad and do not include authentication specifications [22,23]. Ideally, institution-level guidelines would include tutorials for researchers in every stage of project development. These tutorials often do not exist, yet they can be challenging to locate and are not consolidated if available. A previous search of the literature illustrates that there are only a few academic institutions and professional associations that provide written conceptual frameworks for ethically responsible and compliant internet-based research [6]. Further, these frameworks are not applied in scientific settings and do not articulate exactly how to create an ethical and compliant authenticated website [6].

## Purpose

This tutorial provides a case study describing the development of our research team’s interactive health promotion website to address the gap in practical guidelines. Below we detail lessons learned through a solution-focused framework, complete with specific examples from our institution and our research study *denoted in italics.* Our research team, comprised solely of behavioral researchers, set out to create an authenticated website with a blog component to engage research participants assigned to the intervention arm of a randomized controlled trial. We intended to enhance our primary in-person intervention activities by providing an opportunity for research participants to blog about their experiences and engage with others in the intervention arm of the trial by posting informal text entries or uploading pictures and videos. This opportunity needed to be limited to only intervention participants to prevent the threat of cross-contamination (ie, access to intervention content by control participants). It was necessary to develop an authenticated website to create a secure platform that prohibited posts from being visible to participants in the control group or the general public.

Due to the limited technical expertise of our research team (ie, our core research team did not include IT nor data privacy experts), it was critical to get assistance to ensure compliance with IRB and legal recommendations at every stage of website development. Notably, during the feasibility and design phases, our team was presented with a variety of unanticipated security and privacy challenges. As a result, our workflow was dynamic and flexible throughout all phases (feasibility, design, implementation, and maintenance) of the project; we made iterative adjustments to ensure that our activities were both ethical and compliant. We present information in our tutorial to provide behavioral researchers with a roadmap for unforeseen issues that may arise when creating and disseminating a secure research website. Of importance, we discovered that leveraging cross-sector collaboration with an interdisciplinary team was a crucial strategy to adhere to all ethics and compliance standards relevant to our authenticated platform.

Although online resources for creating websites do exist at our large research-based university, specific instructions for authentication and institutional data privacy and security policies are not readily available. We discovered that these instructions also vary by department. For example, at our institution, some departments or schools that conduct research involving community members—rather than with patients in clinical settings or de-identified electronic health record data—may or may not be required to follow the same ethical guidelines for managing PHI/PII that are prescribed by HIPAA. To navigate our institution’s security and privacy rules and regulations, we needed to engage with IRB and legal experts in the formative stages before engaging with IT. The IT team then provided tools and expertise to assist us in building a secure website that met research ethics and compliance standards. Initial conversations led to several months of interdisciplinary collaborations that were vital to understanding specific safeguards for protecting PHI/PII to execute our website project successfully. The goal of this tutorial is to share insights gleaned from these interdisciplinary collaborations. Through our case study, we provide specific considerations and recommendations unique to each phase of our project: feasibility, design, implementation, and maintenance. Lessons learned will be useful to health behavior researchers interested in creating ethically responsible and compliant authenticated websites. Research ethics and compliance guidelines differ by country and, thus, researchers outside of the US may need to amend the following tutorial. However, our tutorial highlights key issues of consideration that apply to website development in any behavioral health research context.

## Feasibility Phase

### Determine Your Website Purpose & Target Audience: Is Authentication Warranted?

The first and most important consideration when conceptualizing a website for your research project is to determine whether or not electronic authentication is needed. It is, therefore, necessary to understand the overall purpose of your website within the context of your research project’s goals. As a general rule, websites that utilize interactive features require more stringent ethical stipulations than websites that provide static site content (ie, “read-only” materials). Authentication is warranted if the goal is to engage research participants in activities that would be covered by institutional confidentiality protections, such as using a social component (eg, blogging, gaming) for a research intervention. Authentication is also warranted to ensure confidentiality and data security if the goal is to observe or collect data from participants rather than provide them with resources. If it is not immediately apparent to your team that authentication is warranted, you must then consider how the website will engage your target audience and determine whether or not the website content should be accessible to the public. Authentication is warranted if your website includes an interactive component and has the potential to display PHI/PII. *This was the case with our website’s interactive blog that allowed research participants to post text and/or pictures, as text could potentially contain identifiable content, and images could include visually identifiable features.* Notably, authentication is also recommended for static websites if the program is accessible to certain research participants at different times, *as was the case with our research design that provided website access to different cohorts at varying time points.*

### Determine Your Institution’s Technical Capacity for Authentication: Is Authentication Feasible?

The next consideration when conceptualizing your website is to work with IT professionals to assess your institution’s technical capacity for authentication and to determine whether ethical and compliant website creation is feasible. First, you will need to gain access to an appropriate web content management system (CMS), like Drupal or WordPress, that you can integrate with authentication systems [24]. *At our institution, Drupal was the only CMS that could be authenticated.* Once you have confirmed that authentication is feasible, you will need to verify if your institution has the technical capacity to create de-identified usernames, which preserves your research participants’ identity and ensures that data privacy is maintained during the authentication process.

### Determine Your Interdisciplinary Team: Is Authentication Supported?

**Figure 1:**
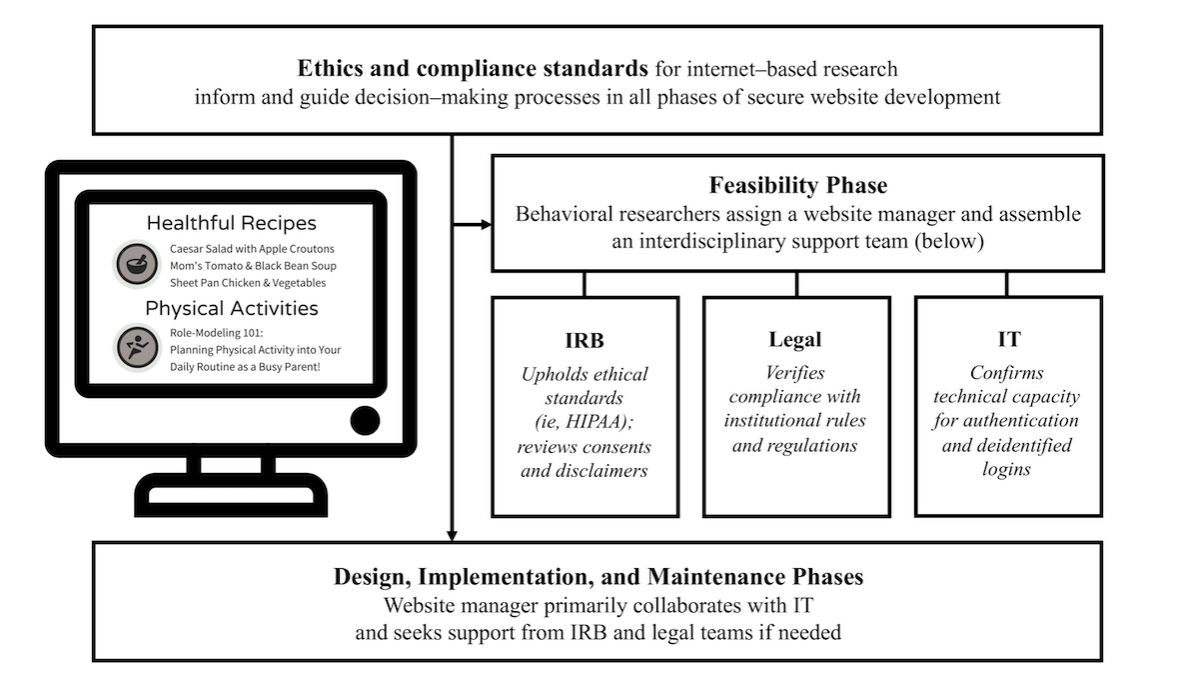
Workflow representing interdisciplinary collaboration required for secure website development in a university research setting. IRB: Institutional Review Board; IT: information technology.

The final consideration when conceptualizing your website is to determine your institution’s ability to support the project throughout all stages of development, from feasibility and design to maintenance and completion. It is crucial to collaborate with an interdisciplinary team from the outset to mitigate any issues that may arise. Support personnel should include a website manager, as well as ethics (ie, IRB), compliance (ie, legal), and technology (ie, IT) experts. *At our university, online tutorials or written guidelines for ethical and compliant website development did not exist, so we reached out to the IRB and legal teams early in the process after reaching out to IT to confirm technical capacity.* See Figure 1 for a workflow representing interdisciplinary collaboration.

#### Website Manager

After you have evaluated the needs of your target audience and technical capacity for authentication, you should appoint a website manager(s). *For example, we chose someone with previous website design and management experience to fill this role.* The website manager(s) should then seek out existing website regulations and ethics and compliance guidelines and become familiar with their institution’s current technical safeguards to protect privacy and anonymity. This person or team will be responsible for understanding authentication procedures as well as determining the content, layout, aesthetics, and navigation tools to be utilized during the initial design phase. All decisions should be made in tandem with recommendations from IT experts, as they are most knowledgeable about your institution’s authentication processes. *In our case, these individuals also guided additional institutional requirements (ie, mandatory branding, simultaneously web and mobile*-*friendly interfaces, accessibility features for those with visual impairments) and other web*-*specific tips (ie, reusable HTML scripts) to help expedite the development process.*

#### Institutional Review Board

The IRB can guide you through the creation of informed consent documents to make sure your website upholds federal and institutional ethical standards to protect human subjects. If your website is not in and of itself an intervention but rather supplements your research, you may want to consider “opt-in/opt-out” disclaimers that allow research participants to choose whether or not they receive access to the website. Regardless of the consent processes that you utilize, make sure to outline the risks and benefits of engaging with your website in your consent form; the presence of this information will be verified and approved by the IRB before study initiation as part of standardized informed consent procedures. *For example, we made it clear to our research participants that to minimize perceived risks, they could access the website and download materials with a de-identified login without having to participate in the interactive blog component.*

#### Legal Experts

In addition to the necessary contact with the IRB before starting human subjects research, it is essential to seek guidance from legal experts to align with institutional rules and regulations. Legal experts can suggest the appropriate informed consent processes to protect your research participants’ anonymity and can suggest additional risk reduction strategies that you should outline in research protocols to align with best practice. Additionally, it is essential to seek advice from research compliance teams that understand institution-specific rules and regulations. *For example, we specifically reached out to internet security analysts, health information compliance officers, and lawyers familiar with HIPAA compliance. At our institution, health information compliance officers are affiliated with the Research Compliance Office, which oversees university-wide ethical and regulatory standards.*

#### Information Technology Experts

During the feasibility phase, your IT team can inform you of existing data security processes and tools to protect PHI/PII. *For example, we specifically reached out to expert website developers within the Office of Information Technology to better understand the available IT tools.* All technical security measures taken must be in alignment with institutional ethics and compliance standards, as these may differ depending on location [6,7,21,25-27]. After IT specialists have confirmed technical capacity and your IRB and legal teams have laid the foundation for creating an ethical and compliant website, you will then primarily seek guidance from IT specialists during subsequent website development phases. Due to your close partnership with IT, we also recommend that you review ethical recommendations created by the Association of Internet Researchers [23] in conjunction with IT personnel before authenticating or launching your website.

## Design Phase

### Understand Existing Authentication Processes

Once you have determined that your website project is indeed feasible, and you have assigned your primary contact(s) for website development processes, you will need to carefully consider how to uphold ethics and compliance standards during the design phase. First, it is crucial to understand the details of existing authentication procedures, which make your website content accessible only to those with preassigned de-identified usernames and passwords. *Of note, we were interested in assigning usernames and passwords to intervention participants, cohort by cohort over time.* More and more institutions are utilizing two-factor authentication (ie, Duo), whereby a one-time password is sent to a second electronic device as another method of verification [28]. Two-factor authentication is an additional level of security used to prevent identity theft and other online fraud (eg, phishing and malware) [9,29]. This added security is particularly crucial for behavioral researchers who have access to PHI/PII and need to protect it, yet the extra security complicates the login process. *As a result, our study staff engaged with study participants by 1) notifying them that they would receive a predetermined username and password via email, 2) emailing them detailed tutorials with images and text describing how to change their passwords (for security purposes) and to log into the website, and 3) allotting additional time and resources during in-person intervention sessions for technical troubleshooting.*

### Select the Correct Web Content Management System

When selecting a CMS, it is important to note that not all CMSs are created equally in terms of capacity to meet ethics and compliance standards for human subjects research. Therefore, selecting the correct web CMS is crucial. *For example, at our institution, four CMSs were available at the institutional level. Two were part of the G Suite for Education (ie, Google Blogger and Google Sites), and two were different university*-*managed configurations of the CMS Drupal (ie, Drupal Lite and Drupal Enterprise). At the time of web development, Drupal Enterprise was the only CMS that allowed for authentication. While multiple systems were available at an institutional level, our department had its own IT team and communications team available for support, so we needed to interface with several individuals to select the system that met ethics and compliance standards*—*Drupal Enterprise.* In addition to confirming the capacity for password-protected logins, we recommend that you confirm with the support team that your selected CMS will enable you to review and approve, or reject, all content posted by research participants before being visible to others. This extra security measure may prevent inappropriate exposure of PHI/PII to participants and hackers alike.

### Choose a Suitable Website Host

Once you have determined the appropriate CMS, you will have to decide which department within your larger institution can host your authenticated website. *For our research team, the academic department with whom we were associated could not host authenticated websites for research, so we reached out to our administrative umbrella organization that oversees multiple academic departments within the university. The Drupal support team affiliated with this organization agreed to host our website.* Of note, once you have selected an adequate host, it is vital to determine the type and breadth of support you will receive from your host, as the turnaround time will directly impact the length of the development phase. If assistance is not timely or adequate, you will need to consider this when determining the appropriate team member to manage your website. *In our case, the Drupal support team provided prompt technical assistance throughout our project.*

### Select the Appropriate Internet Account

To create de-identified logins for your research participants, you will need to set up individual internet accounts. Similar to CMS and website hosts, internet account types vary considerably. *For example, at our institution, there are a variety of internet accounts (ie, sponsored accounts; proxy accounts; departmental/organizational accounts), each with differing levels of access and stipulations for expiration.* To determine which account is best, you must first consider whether your research participants already have an affiliation with your organization or whether they will need a separate account that is HIPAA compliant. *For example, guest accounts at our institution typically use the first part of a participant’s email, which potentially could contain PHI/PII and thus should not be used.* You will then need to determine how long your participants will need to access your website and make sure that the account can be discontinued at an appropriate time. Next, you should consider the level of access needed. *Most internet accounts at our institution are created with access to other services, such as the G Suite, which provides access to a variety of Google Cloud computing tools like Gmail, Docs, Drive, and Calendar. For our project, we only needed to create usernames for our research participants, not an additional email account, so departmental accounts were best suited for our website project because we could manually “opt-out” of G Suite. These usernames were then linked to Drupal Enterprise on the backend, granting login access to each user.* Of note, regardless of the type of internet accounts you ultimately choose, it is essential to avoid using identifiable information, as it could be visible to your IT help staff if and when they assist you with creating or changing usernames and passwords.

## Implementation Phase

### Develop and Beta Test

After designing your website, you will need to develop and beta test it before granting access to your research participants. You should test the site and the desired features to ensure that they meet your project’s goals. *For example, during this phase, we tested the authentication process with temporary usernames and made sure that all website content was accessible from multiple devices (eg, desktops, laptops, tablets, mobile phones). We also tested all hyperlinks and made sure that the blog posts were only visible to research participants once our web manager approved them.*

### Create Internet Accounts/Deidentified Logins

After beta testing and documenting participant consent, choose the appropriate internet account type that will allow you to create individualized, de-identified logins for all participants. Then set a reasonable expiration date (eg, the length of the intervention) and make sure to link the de-identified usernames to your CMS on the backend. To facilitate the process, you may want to consider a one-time password reset for all newly created accounts before sharing the account information with research participants. *This extra step worked well for us, given that our institution required that all initial passwords be changed within 24 hours; a second password reset was completed at the research participants’ leisure. For our website project, we created pseudonyms for our research participants and stored all internet account information on a secure server, accessible only to a limited subset of our research team who had IRB approval.*

## Maintenance Phase

### Maintain Your Website

Once you have gone live with your site, make sure you visit the site regularly. *In our case, we developed separate pages of content in alignment with our monthly in-person sessions because we wanted research participants to access the online material on a month-to-month basis. Therefore, regular biweekly website maintenance was crucial to align with our research project’s overall goals.* Indeed, ensuring that your website is properly maintained is not only necessary for overall functionality but also to secure the site from unintended breaches of confidentiality. *Frequent monitoring was especially crucial for our website project, given that our platform did not contain built-in alerts when someone posted on the blog. Although we approved all posts before they went live, unintended security breaches could have occurred if we did not conduct regular maintenance.*

### Project Completion

Upon project completion, you will need to complete three critical steps to ensure data privacy. First, confirm the expiration of the de-identified logins. *At our institution, the IT department sent an email notifying the website manager of upcoming internet account expirations*. Second, at the end of the intervention, double-check to make sure all de-identified logins are disconnected from your CMS. These precautions prevent users from accessing the site after completion of the intervention. *Of note, we were able to disconnect users from Drupal without deleting blog posts, which served useful when analyzing usage after completion*. Finally, make sure that all participant information associated with de-identified usernames is stored on a secure server and only available to approved research personnel for a predetermined length of time denoted in your research protocol. *Per our IRB and grant funder’s rules and regulations, we were instructed to keep digital copies of all participant data on a secure server for seven years after completion of the randomized controlled trial.* These three steps are vital to adhere to ethics and compliance standards, particularly if your compliance office ever audits you.

## Discussion

### Takeaways for Authenticated Website Development and Implementation

Despite the increasing trend of developing digital platforms to deliver or support health behavior research, practical or applied ethics and compliance guidelines for research-based website development are lacking in the literature, and institution-specific tutorials are often not readily available. As depicted in our case study, designing and implementing an authenticated digital platform that aligns with best practices for human subjects research requires time and sufficient resources. Therefore, we present this case study protocol with suggested guidelines as a roadmap for research teams. Although we linearly present our considerations, it is essential to note that website development is an iterative process, and you may need to adjust your timelines throughout all stages of design and implementation. We also highly recommend that you consider the above recommendations well in advance of going live with your website, and that you collaborate closely with IRB, legal, and IT experts early in the process. These early partnerships are vital to the success of creating an authenticated website that abides by ethics and compliance standards.

In summary, lessons learned from our applied case study highlight three overarching considerations future researchers must be aware of before launching health behavior research-based authenticated websites. First, behavioral researchers must be mindful that when they ask participants to be cocreators or sharers of intervention materials through blogging or uploading content to the website, it is their responsibility to protect participants’ anonymity and privacy through the provision of an authenticated website. Second, when striving to create authenticated websites that align with ethics and compliance standards, behavioral researchers must expect the unexpected. Due to unanticipated challenges, we needed extra time to learn the ins and outs of ethically responsible and compliant website authentication outlined above. While our paper has filled a gap in the literature by providing researchers with an applied tutorial for developing an authenticated website, we acknowledge the novelty of the field of digital health research and know that our proposed best practice recommendations will likely morph with changes in technology-based systems. We also acknowledge that authentication processes, technological capacities, and availability of ethics (ie, IRB), compliance (ie, legal), and technical (ie, IT) support teams may differ from institution to institution, so we present these recommendations as an initial template for best practices. Third, behavioral researchers should note that although programming considerations and design phases may differ by project and institution, the foundation of all website-oriented decision making must be grounded in ethical principles. Thus, to conduct ethical and compliant research, interdisciplinary teams must rigidly prioritize the protection of participants’ privacy and confidentiality in all phases of website design and implementation.

### Future Considerations

This paper provides behavioral researchers with an applied tutorial for secure website development and implementation. Project takeaways and lessons learned from our interdisciplinary collaborations may assist future researchers in designing secure websites that could be used as a standalone intervention or as an intervention supplement. We recommend that colleagues consider developing individualized protocols for their respective universities or institutions since there are few published evidence-based guidelines for ethical and compliant website development in behavioral research. Moreover, authentication processes and technologies differ from institution to institution, and researchers must consider this variance. Publishing applied tutorials or guidelines will advance the science of ethically responsible and compliant internet-based behavioral research, as the dissemination of institutional guidelines will provide opportunities for knowledge to be shared across sectors.

Furthermore, we acknowledge that although ensuring security is vital to technology-based behavioral research, researchers may not always be the most knowledgeable when it comes to creating secure and authenticated websites. Behavioral researchers should leverage interdisciplinary collaborations to be compliant with ethical standards and institutional rules and regulations. To ensure project success, we promote interdisciplinary team science and encourage behavioral researchers to engage in a comprehensive dialogue with IRB, legal, and IT professionals throughout all phases of website development.

## Abbreviations

CMS: Content Management System
HIPAA: Health Insurance Portability and Accountability Act
IRB: Institutional Review Board
IT: Information Technology
PHI: Personal Health Information
PII: Personally Identifiable Information
